# Dizziness, but not falls rate, improves after routine cataract surgery: the role of refractive and spectacle changes

**DOI:** 10.1111/opo.12243

**Published:** 2015-11-09

**Authors:** Elvira Supuk, Alison Alderson, Christopher J. Davey, Clare Green, Norman Litvin, Andrew J. Scally, David B. Elliott

**Affiliations:** ^1^ Bradford School of Optometry and Vision ScienceUniversity of BradfordWest YorkshireUK; ^2^ Ophthalmology DepartmentBradford Teaching Hospitals Foundation TrustWest YorkshireUK; ^3^ Faculty of Health StudiesUniversity of BradfordWest YorkshireUK

**Keywords:** astigmatism, cataract surgery, dizziness, falls, multifocals, refractive correction

## Abstract

**Purpose:**

To determine whether dizziness and falls rates change due to routine cataract surgery and to determine the influence of spectacle type and refractive factors.

**Methods:**

Self‐reported dizziness and falls were determined in 287 patients (mean age of 76.5 ± 6.3 years, 55% females) before and after routine cataract surgery for the first (81, 28%), second (109, 38%) and both eyes (97, 34%). Dizziness was determined using the short‐form of the Dizziness Handicap Inventory. Six‐month falls rates were determined using self‐reported retrospective data.

**Results:**

The number of patients with dizziness reduced significantly after cataract surgery (52% vs 38%; χ^2^ = 19.14^,^ *p* < 0.001), but the reduction in the number of patients who fell in the 6‐months post surgery was not significant (23% vs 20%; χ^2^ = 0.87, *p* = 0.35). Dizziness improved after first eye surgery (49% vs 33%, *p* = 0.01) and surgery on both eyes (58% vs 35%, *p* < 0.001), but not after second eye surgery (52% vs 45%, *p* = 0.68). Multivariate logistic regression analyses found significant links between post‐operative falls and change in spectacle type (increased risk if switched into multifocal spectacles). Post‐operative dizziness was associated with changes in best eye visual acuity and changes in oblique astigmatic correction.

**Conclusions:**

Dizziness is significantly reduced by first (or both) eye cataract surgery and this is linked with improvements in best eye visual acuity, although changes in oblique astigmatic correction increased dizziness. The lack of improvement in falls rate may be associated with switching into multifocal spectacle wear after surgery.

## Introduction

Falls are the major cause of death and non‐fatal injuries in the elderly.^1^, ^2^ They are also relatively common, with at least a third of community‐dwelling, healthy adults aged 65 years and over falling once a year or more.^1^, ^2^ Falls in older adults are not random, chance events or ‘accidents’, but typically multifactorial and linked to geriatric syndromes^3^, ^4^ and most epidemiological studies have shown that visual impairment is a significant and independent risk factor for falls with an average odds ratio of 2.0.^5^ In addition, clinical audit studies have reported that many older adults who attended emergency clinics because of a fall or who had undergone hip fracture surgery had visual impairment, of which about one‐third was correctable by cataract surgery.^6^, ^7^

These studies suggest that providing cataract surgery to older people at risk of falling would lead to reductions in falls rates. However, although two open‐design intervention studies of cataract surgery found significant improvements in falls rates after cataract surgery,^8^, ^9^ cohort studies and randomised controlled trials provide much more equivocal results.^10^, ^11^, ^12^ Indeed, Deandrea *et al*.^13^ concluded that there was no evidence that cataract surgery reduced falls rate after combining the data from the two randomised controlled trial studies^11^, ^12^ in a meta‐analysis. The results from large‐scale assessments of the effect of cataract surgery on injurious falls have also been equivocal.^14^, ^15^

Self‐reported dizziness was also included as a principal outcome measure in this study. Dizziness is highly prevalent in the older population,^16^ is linked to falls^13^, ^17^, ^18^ and may be increased with poor vision.^19^, ^20^, ^21^

We hypothesised that there are some factors associated with cataract surgery that lead to a relatively greater risk of falling (and increased dizziness) which may in some circumstances offset the reduction in falls risk (and dizziness) due to improvements in visual function. These could include the following:

- Increased anisometropia after first eye surgery.^14^, ^22^
- Adaptation problems to large changes in refractive correction.^23^ These include spectacle magnification and astigmatic distortion changes increasing trip risk on steps and stairs^24^, ^25^ and requiring adaptation of the vestibulo‐ocular reflex gain. Patients can complain of their visual world appearing to ‘swim’ until adaptation occurs.^24^
- Switching to multifocal spectacles.^26^, ^27^, ^28^
- Increased confidence leading to greater outdoor activities and increased fall risk.^23^

These factors were assessed and multivariate logistic regression analyses were used to determine whether they had a significant independent effect on post‐operative falls rates and self‐reported dizziness.

As far as we are aware, this is the first study to attempt to determine why cataract surgery does not improve falls rate as much as expected and the first to evaluate the effect of cataract surgery on dizziness.

## Methods

### Study design and participants

Participants were recruited for this cohort study from the cataract waiting lists of two hospitals in the UK, Bradford Royal Infirmary and Yorkshire Eye Hospital, between July 1, 2012 and July 31, 2013. All patients 65 years and older who were listed for routine phacoemulsification with a monofocal IOL during this period were sent details about the study (~1240). The study was approved by the East of England NHS Research Ethics Committee and adhered to the tenants of the Declaration of Helsinki. All participants gave written, informed consent to participate in the study and for the research team to access their hospital medical records and contact their optometrist for relevant information regarding their refractive correction.

### Procedures

Close to the surgery date participants were sent a list of questions requesting information regarding outdoor activity levels (everyday, 1–2 times per week, 1–2 times per month, seldom or never) and type of spectacles worn for walking (none, single vision, progressive addition or bifocals). Data regarding the participants' age, sex, general health status, the number and type of prescribed medications and their pre‐ and post‐operative habitual refractive correction and habitual visual acuity (i.e. with the spectacles they were usually wearing for distance tasks) were obtained from the participants' medical records. Any missing data were followed up by telephone calls to the participant and/or their optometrist.

#### Dizziness assessment

The most commonly used and accepted questionnaire to quantify the impact of dizziness on everyday life is the Dizziness Handicap Inventory (DHI) yet despite this its structural validity is not established.^29^ In this study, participants were sent a copy of the short form of the dizziness handicap inventory (DHIsf), which has been validated using Rasch analysis.^30^ The DHIsf comprises 13 questions, each with a yes/no answer and a participant with a score of 13 has no handicap from dizziness whereas a score of zero would indicate extreme handicap. Participants were asked to complete the DHIsf with regards to any dizziness they had suffered in the previous month. Patients were sent the DHIsf approximately 1 month prior to surgery and 1 month post surgery (after the 2nd eye for those that had surgery on both eyes).

#### Falls assessment

Given the short waiting times for NHS cataract surgery at the time, patients could only be recruited to the study a relatively short period prior to their surgery (median 25 days, range 14–40 days). To obtain an assessment of pre‐operative self‐reported falls over a larger period, we retrospectively asked if patients had fallen within the last 6 months. A fall was defined as ‘an unexpected event in which the participants come to rest on the ground, floor, or lower level’.^31^ All patients that reported falling were asked to give further information including the number of falls that had occurred and whether they were wearing spectacles at the time of each fall. In an attempt to improve the accuracy of the falls data, participants were also sent falls diaries in the period after consenting to participate in the study and before surgery (median 25 days, range 14–40 days).

Self‐reported 6‐month falls data were collected in the same way post‐operatively: participants were sent monthly falls diaries for completion for 1–2 months (equivalent to the number of completions pre‐surgery) and after 6 months, they were sent the same questionnaire requesting information regarding the occurrence of falls in the previous 6 months. The response to the 6‐month retrospective falls question was checked against the falls diary information and any inconsistencies were investigated (although the falls diary information was only a useful check for the situation of a reported fall in the falls diary versus no fall in the 6‐month retrospective information and this did not occur). The data reported are the self‐reported 6‐month falls data.

### Statistical analysis

A target sample size of 280 was calculated using Peduzzi and colleagues^32^ formula of *N* = 10 *k*/p, where *k* is the number of covariates accounted for and p is the likely proportion of positive cases (falls rate in this study and taken to be 25%). Data analysis was carried out using STATA, version 13.1. For analysis all Snellen visual acuity (VA) measurements were converted to logMAR. The participant's habitual refractive correction (i.e. their spectacles worn when walking) was converted into power vector format to enable comparison of pre and post‐operative data.^33^ As we were interested in the overall change in both spherical correction and astigmatism, but not the direction of the change (i.e. we assumed that in terms of their effect on dizziness and falls, a 6.00DS reduction in hyperopic correction would have similar effects to a 6.00DS myopic reduction and a 1.00DC swing towards against‐the‐rule astigmatism would be similar to a 1.00DC swing towards with‐the‐rule astigmatism), the absolute value of the changes due to surgery in mean sphere equivalent and the vector values of astigmatism J_0_ and J_45_ were used in the analyses. Changes in refractive correction from second eye surgery were used in the analyses from patients who underwent surgery in both eyes as falls were assessed after second eye surgery for those patients.

Normality of continuous data was determined using the Kolmogorov‐Smirnov test. Age, dizziness, number of medications, number of chronic conditions and vision data were not normally distributed and therefore they are described in terms of medians and inter‐quartile ranges (IQR). Differences between demographic data for included and excluded participants were analysed using the Mann–Whitney *U* test for continuous data and the chi‐square test for categorical data.

A comparison of the DHIsf score before and after cataract surgery was carried out using the Wilcoxon signed rank test for dependent samples. As the dizziness score data were highly skewed, participants were dichotomised into those who scored 13 on the DHIsf as having no handicap from dizziness, and those who scored <13 on the DHIsf as having some level of handicap from dizziness. Changes in prevalence of dizziness and falls pre and post‐surgery were analysed using McNemar's test. As the activity levels data were skewed, participants were dichotomised into those who were active (*n* = 248; outdoor activity at least 1–2 times per week) and those who were inactive (*n* = 39; outdoor activity 1–2 times per month or less). Visual acuity and refractive correction data were assigned to the ‘best’ and ‘worst’ eye (rather than the operated and non‐operated eye) as binocular visual function is typically related to vision in the best eye.^34^ Multivariate logistic regression models for post‐operative self‐reported falls and dizziness (both dichotomous, falls or not, dizziness or not) were developed that included age and sex and any significant medical factor. Any visual and/or refractive factor that showed a univariate logistic regression *p*‐value of <0.10 were then entered into the model to produce final models of independent risk factors for both post‐operative self‐reported dizziness and falls.

## Results

**Figure 1:**
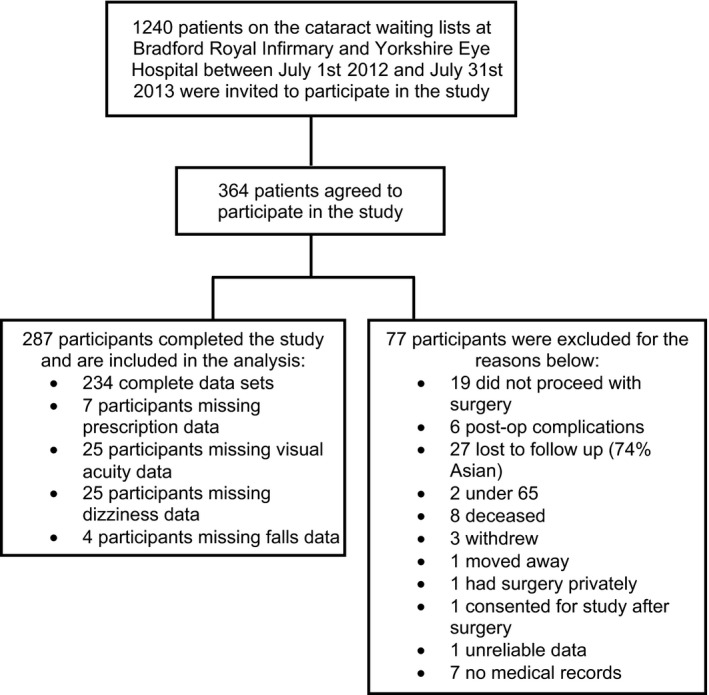
A breakdown of the reasons for participant exclusion.

Three hundred and sixty‐four patients indicated an interest in participating. Seventy‐seven were excluded from the study and/or analysis with 287 (79%) completing the study. This is similar to the recruitment in the earlier UK cataract surgery studies.^11^, ^12^ A breakdown of the reasons for exclusion is shown in *Figure* 1. There was no significant difference in age (U = 10245, *p* = 0.33) or sex (χ^2^ = 0.11, *p* = 0.80) between those included (age 77 years, 55% female) and those excluded (age 74 years, 53% female) from the study. Of the 287 patients completing the study (mean age 76.5 ± 6.3 years; 55% females; 93% Caucasian), 81 (28%) had routine cataract surgery in the first eye, 109 (38%) in the second eye and 97 (34%) had surgery in both eyes. The latter group were those patients in whom there was <6 months between first and second eye surgery (median 57 days, IQR 43‐81 days) so that we were unable to collect 6‐month falls data between surgeries. For these participants, a post‐operative falls rate was collected for the 6‐month period after their 2nd eye surgery.

Median (and IQR) refractive correction and habitual VA data before and after cataract surgery for the operated and non‐operated eye are provided in Table 1. The number of patients wearing spectacles for distance viewing was reduced after surgery (from 196/287, 68% to 147/285, 52%; Fishers exact test, *p* < 0.0001).

**Table 1:** Median (inter‐quartile range) pre and post‐operative absolute values of refractive correction (N = 280) and habitual visual acuity (N=262) before and after cataract surgery in the operated and non‐operated eye

	Operated Eye		Non‐Operated Eye (1st eye surgery)		Non‐Operated Eye (2nd eye surgery)	
	Pre‐Op	Post‐op	Pre‐Op	Post‐Op	Pre‐Op	Post‐Op
MSE (D)	1.25 (0.00–2.88)	0.0 (0.00–0.50)	1.38 (0.00–2.25)	0.00 (0.00–1.34)	0.00 (0.00–0.50)	0.00 (0.00–0.38)
J_0_ (D)	0.13 (0.00–0.48)	0.00 (0.00–0.31)	0.13 (0.00–0.48)	0.00 (0.00–0.28)	0.00 (0.00–0.19)	0.00 (0.00–0.39)
J_45_ (D)	0.07 (0.00–0.27)	0.00 (0.00–0.13)	0.04 (0.00–0.19)	0.00 (0.00–0.08)	0.00 (0.00–0.19)	0.00 (0.00–0.16)
Habitual VA	0.30 (0.20–0.40)	0.10 (0.00–0.24)	0.20 (0.10–0.28)	0.24 (0.10–0.80)	0.20 (0.00–0.26)	0.10 (0.00–0.24)

Changes in the non‐operated eye are typically due to changes in spectacle wear.

MSE, mean spherical equivalent in dioptres; J_0_ and J_45:_ Vector values of astigmatism in the ordinal and oblique meridians respectively; Habitual VA, monocular visual acuity (in logMAR) measured with the patients' own distance spectacles if worn.

The self‐reported 6‐month falls rate remained similar before and after cataract surgery (χ^2^ = 0.87, *p* = 0.35), with 66 of 287 (23%) of the participants reported falling in the 6 months prior to surgery compared to 56 of 283 (20%) that reported falling in the same period after surgery. Levels of activity were similar before (86% active) and after surgery (83% active).

The median DHIsf score improved significantly following cataract surgery from 12 (IQR: 9–13) to 13 (IQR: 11–13), *z* = −13.38, *p* < 0.001, indicating a reduction in dizziness. In the month prior to surgery 52% of participants suffered some form of handicap due to dizziness, whereas in the month after surgery this figure was reduced to 38% (χ^2^ = 19.14^,^ *p* < 0.001). This was similar for surgery on the first eye or both eyes, but the improvement was not significant for second eye surgery (first eye surgery, 49% vs 33%, *p* = 0.01; second eye surgery 52% vs 45%, *p* = 0.68; surgery on both eyes, 58% vs 35%, *p* < 0.001).

The most parsimonious multivariate logistic regression models showing significant, independent medical and visual/refractive factors are shown in Tables 2 and 3 for post‐operative falls and dizziness respectively. Age and sex were initially included in the post‐operative falls model, but were not significant when pre‐operative falls were included (age, *p* = 0.47; sex, *p* = 0.99) and their inclusion did not substantially affect the influence of the other variables that we did include. Medical factors such as arthritis were weakly associated with post‐operative falls (OR 1.84, CI 0.96–3.52; *p* = 0.07) and post‐operative dizziness was strongly associated with falls (OR, 3.34, CI 1.78–6.26; *p* < 0.0001), but pre‐operative falls was a much stronger risk factor and acted as a proxy for multifactorial risk factors including age. Post‐operative dizziness was retained in the final model (despite a *p*‐value of 0.10) as it was an important predictor of interest and may be on the causal pathway between vision changes and falls. Changing into multifocals post surgery increased falls risk significantly (OR = 3.56, CI 1.34–9.43, *p* = 0.011; also see Table 4).

**Table 2:** Final multivariate logistic regression model containing independent risk factors for falls in the 6 months post‐surgery (*n* = 265)

	Adjusted (multivariate)		Non‐adjusted (univariate)^a^	
	OR (95% CI)	p‐value	OR (95% CI)	*p*‐value
Pre‐operative falls	7.28 (3.48–15.21)	<0.0001	8.74 (4.56–16.76)	<0.0001
Post‐operative dizziness	1.83 (0.89–3.74)	0.10	3.34 (1.78–6.26)	<0.0001
Change into multifocal spectacles	3.56 (1.34–9.43)	0.011	2.52 (1.09–5.85)	0.03
Change from multifocal spectacles	1.81 (0.64–5.15)	0.27	1.60 (0.64–4.02)	0.32

Adjusted (within the model) and non‐adjusted univariate odds ratios (OR) are shown with 95% confidence intervals (CI) and *p*‐values. The likelihood ratio chi‐squared value for the model was 49.2 (*p* < 0.0001) with pseudo *R* ^2^ = 0.19.

a Other univariate odds ratios (*p*‐value) for factors not included in the final multivariate model included age 1.06 (0.017), sex 1.26 (0.45), walks outside 0.80 (0.55), number of chronic conditions 1.23 (0.052), number of medications 1.07 (0.13), arthritis 1.84 (0.065), sedative use 1.62 (0.25), best visual acuity post surgery 0.76 (0.76), change in best visual acuity 0.55 (0.45), change in anisometropia 0.85 (0.28), change in mean sphere equivalent 0.91 (0.44), change in J_o_ 0.71 (0.57), change in J_45_ 0.77 (0.69).

**Table 3:** Final multivariate logistic regression model containing independent visual risk factors for dizziness in the month post‐surgery (*n* = 262)

	Adjusted (multivariate)		Non‐adjusted (Univariate)^a^	
	OR (95% CI)	*p*‐value	OR (95% CI)	*p*‐value
Pre‐operative dizziness	12.08 (5.80–25.16)	<0.0001	14.42 (7.48–27.79)	<0.0001
Age	1.07 (1.01–1.13)	0.013	1.07 (1.03–1.11)	0.001
Sex (female)	1.90 (0.96–3.76)	0.065	1.86 (1.12–3.09)	0.016
Number of medications	1.17 (1.05–1.31)	0.005	1.20 (1.10–1.31)	<0.0001
Change in best eye habitual visual acuity	0.14 (0.02–0.83)	0.03	0.23 (0.06–0.90)	0.03
Change in best eye J_45_	6.60 (1.36–32.07)	0.019	7.87 (2.26–27.34)	0.001

Adjusted (within the model) and non‐adjusted univariate odds ratios (OR) are shown with 95% confidence intervals (CI) and *p*‐values. The likelihood ratio chi‐squared value for the model was 103.7 (*p* < 0.0001) with pseudo *R* ^2^ = 0.32.

a Other univariate odds ratios (*p*‐value) for factors not included in the final multivariate model included walks outside 0.30 (<0.0001), number of chronic conditions 1.22 (0.03), arthritis 1.58 (0.12), sedative use 2.98 (0.007), best visual acuity post surgery 37.46 (<0.0001), change in anisometropia 1.16 (0.16), change in mean sphere equivalent 1.06 (0.52), change in J_o_ 2.65 (0.03), Change into multifocal spectacles 1.27 (0.56), Change from multifocal spectacles 1.17 (0.71).

**Table 4:** The post‐operative falls rate of patients who changed either into or out of multifocal spectacles (bifocals and progressives) after cataract surgery compared to those that continued with multifocal wear or continued with their own distance single vision spectacles or no spectacles

Post‐op spectacle wear	*N*	Falls rate
Into multifocals	30	30%
Continued with Multifocals	62	23%
Discontinued multifocals	53	15%
Continued with single vision spectacles or without spectacles	133	17%

Table 3 indicates that dizziness was present in patients who suffered from dizziness pre‐surgery, with increasing age, with the number of medications and with greater changes in oblique astigmatism in the refractive correction. Post‐operative dizziness was reduced for patients with larger changes in best eye habitual visual acuity.

## Discussion

We found a substantial falls rate both before and after cataract surgery and identified risk factors relating to spectacle management. The multifactorial logistic model with post‐operative falls as the outcome measure showed associations with pre‐operative falls and changes in spectacle type (into multifocal lens wear; Table 2). There were no significant associations with post‐operative VA in the best eye, change in mean spherical equivalent refractive correction, change in astigmatic correction (J_0_ or J_45_), change in anisometropia (all *p* > 0.10) or post‐operative activity levels. However, the number with large refractive changes in the operated eye were relatively small (over 4.00D: 33/283, 12%) and Cummings^23^ found an increased falls rate with large change in spectacle correction (although the influence of myopic, hyperopic and astigmatic changes were all combined in their report), so further studies with larger numbers of high refractive corrections before surgery may be useful. Activity levels were similar before and after surgery, with 19 participants becoming inactive after surgery when active before surgery. This was typically due to ill health (e.g., arthritis, hip and knee problems). However, of the small number who greatly increased activity after surgery, all ten had no falls before surgery, but four fell after surgery. It is possible that this increased walking about outside the home put the patient at increased risk of falls, particularly while adapting to the new level of vision.^23^ However, the numbers in the subsample are small and a larger sample study is required.

The association between falls and changes into or out of multifocal spectacles are shown in Table 4. The falls rate in patients switching into multifocals is double (30%) that of those patients who discontinued multifocal wear (15%). This is in agreement with much of the literature, which suggests that multifocals are a risk factor for falls^26^, ^28^ due to blur in the lower visual field (both bifocals and progressive addition), variable areas of vestibulo‐ocular reflex gain^35^ peripheral distortion in progressive addition lenses and diplopia and image jump at the reading segment edge in bifocals.^27^ One area particularly open to change is the correction of ametropia between surgeries. Patients who wore multifocals before first eye surgery and after second eye surgery, but no spectacles in between surgeries (N = 12, falls rate = 33%) would have needed to adapt to not wearing multifocals after first eye surgery (median time of 57 days, IQR 43–81 days; this seems sufficiently long for adaptation to the lack of spectacles to have occurred in most patients)^36^ and then re‐adapt to wearing them after second eye surgery. This would therefore include two adaptations of the vestibulo‐ocular reflex gain, which is variable in multifocals.^35^ The number of patients in this comparison are small and this needs further study.

Self‐reported dizziness was greater in females and patients with multiple medications (Table 3) and this is similar to earlier findings.^21^, ^37^, ^38^ The prevalence of dizziness depends on the population studied and the definition of dizziness used.^21^, ^39^, ^40^, ^41^ Our dizziness prevalence figures are high at 52% pre‐operative and 38% post‐operative and this likely highlights the wide definition used (anybody indicating dizziness to any one of the 13 questions of the DHIsf) and the older age and poor pre‐operative vision of our participants. Given that several studies have shown a strong association between dizziness and reduced quality of life,^40^, ^42^ the significant reduction in dizziness due to cataract surgery could be important. The need for cataract surgery is typically determined by the reduction in the desired lifestyle caused by poor vision due to cataract.^43^ Although this is typically thought to mean everyday tasks that are reliant on vision, such as driving, seeing faces and reading, this study suggests that dizziness could also be a consideration. Dizziness is multicausal, but even in patients with vestibular disease causing dizziness, appropriate treatment of visual problems can be beneficial.^44^ Larger changes in VA (logMAR) reduced the risk of post‐operative dizziness (OR 0.14, Table 3) and the improvement in dizziness due to surgery is presumably due to the improvement in VA and possibly linked with improvements in postural stability.^45^, ^46^ In addition, greater changes in J_45_, the vector representing oblique astigmatism, were a risk factor for post‐operative dizziness (OR 5.2, Table 3). This is not surprising given that astigmatic correction can lead to distortions in how patients perceive in 3‐D space^47^, ^48^ and oblique astigmatism is known to produce the greatest problems of distortion^25^ and difficulties in adaptation.^36^ The strong link between post‐operative falls rate and dizziness symptoms (OR, 3.17, *p* = 0.002) was expected and has been suggested by other studies.^19^, ^20^, ^21^ This suggests that those visual and refractive factors influencing dizziness may also have an indirect influence upon falls rates. In this way falls risk may be reduced due to the reduction in dizziness caused by improved VA and may be increased by changes in oblique astigmatic refractive correction.

The study was limited in several ways. The falls data were self‐reported recall from the previous 6 months and accurate retrospective assessments of falls are difficult due to poor memory recall of older patients^49^ in addition to their self‐reported nature and difficulty in defining exactly what constitutes a fall.^31^ Monocular VA data were taken from clinical records and it would be preferable to measure binocular VA plus contrast sensitivity, visual field and stereoacuity using standardised protocols. Outdoor activity levels were taken from a simple question about the extent of outdoor activity per month and preference would be for a more detailed questionnaire assessment and/or perhaps pedometer measurements. Finally, the study has highlighted several areas that would benefit from data collection from a larger sample of pre and post‐operative cataract surgery patients and these include patients with large ametropic changes and different multifocal wearing patterns of patients undergoing surgery on both eyes.

In summary, this study found that dizziness was reduced by cataract surgery and this was linked with improvements in best eye VA, but increased by changes in oblique astigmatic correction. This needs to be investigated further to determine whether dizziness should be a consideration in the decision of whether to perform cataract surgery. We found no improvement in falls rate with routine cataract surgery. This is probably linked to the relatively good pre‐operative VAs and possibly to too many patients switching to multifocal spectacle wear post‐surgery. This suggests that to maximise the potential for cataract surgery to improve falls rates, patients should be appropriately warned of the potential adaptation problems after surgery, particularly if they had a large change in oblique astigmatism and/or have switched to multifocal wear. In between first and second eye surgeries, multifocal wearers could consider wearing updated multifocals rather than go without spectacles if the intention is to continue multifocal wear post second eye surgery.

## Grant support

This work was supported by The Dunhill Medical Trust (grant number SA14/0711).

## Financial disclosures

No conflicting relationship exists for any author.
